# MousebreedeR: A novel software to assist in the design of breeding schema for complex genotypes of experimental organisms

**DOI:** 10.21105/joss.06474

**Published:** 2024-06-04

**Authors:** Mike Sportiello, David J. Topham

**Affiliations:** 1 Center for Vaccine Biology and Immunology, University of Rochester Medical Center, Rochester, NY 14642, United States of America; 2 Medical Scientist Training Program, University of Rochester Medical Center, Rochester, NY 14642, United States of America

## Abstract

With the advent of gene editing playing a regular role in producing new experimental models, the need to get the appropriate allele at each respective locus in a model organism is now common. Instead of simple “knockout” vs “wildtype” mouse experiments, for example, it is common for there to be a gene of interest that is either floxed (surrounded by known genetic sequence that allow for a gene to be knocked out when coexpression of the cre protein is expressed) or not floxed depending on the experimental group, another gene that marks cells of a certain phenotype such as a fluorescent reporter, a cre expression cassette to flox the gene of interest out, and more. While it is often obvious how to efficiently breed experimental organisms to obtain one locus of interest, once three or more loci are involved this becomes difficult to assess. MousebreedeR is a free and open source web-based shiny app and command line R package that takes desired mouse genotypes as well as the genotypes of potential mouse breeders that the user already has on hand as inputs, and delivers efficient breeding schema as the output. In addition, it supplies the user with the probability of producing each potential pup, the probability that no pups with the desired genotype are born in a litter, and more. We have also supplied code that assists the user in plotting these tables to help them visualize crosses of interest and make decisions in their breeding.

## Statement of need

Efficient breeding is a scientific imperative: with a limited period of fertility in experimental organisms, users may only have limited attempts with organisms of desired genotypes (Handelsman et al., 2020; Murray et al., 2010). Taking into account both gestational time and the necessary time it takes to wean pups off their mother’s milk, an inefficient cross can set the user back months in the case of a female pregnant with the non-optimal male’s offspring. Furthermore, as most research centers pay *per diem* costs, inefficiency is a large direct cost for many experimentalists. Individual cages often cost hundreds of dollars per year just in maintenance fees, not even accounting for the cost of extra rounds of genotyping (requiring PCR primers, PCR mastermix, gels, dye, etc) or for the time dedicated to the project by employees. Most importantly, ethical and animal welfare concerns also arise as inefficient breeding results in the mass euthanasia of non-desired animals as well as ear/tail-clipping required for genotyping, which may cause animals pain and distress.

While the breeding schema to obtain a litter of full knockout mice from one wildtype/wildtype parent and one knockout/knockout parent is straightforward, this is not the case for the situation where one has 4 alleles at 4 loci in 4 separate mice, which all need to co-occur in the same mouse for a given planned experiment, for example. Furthermore, to our knowledge, no current software exists that can quantitatively assist the user in creating their breeding schema. Indeed, if there are 2 alleles at each locus, when attempting to make a genetically marked, inducible, cre-lox model with T cell specificity as our lab was doing and which prompted us to create this software, 81 possible combinations exist (3 (AA, Aa, and aa) to the 4th power). With 81 possible males and 81 possible females to mate, 6561 possible pairings exist (81 * 81). Use of the mousebreedeR software to optimize that breeding schema has been most helpful.
